# A Complete Programme of Child Welfare

**Published:** 1921-08-06

**Authors:** 


### A Complete Programme of Child Welfare.

No department of social activities has developed more rapidly than what is known as "Child Welfare." Even as lately as twenty years ago the provision of trained midwives and of a few more or less ill-managed crèches satisfied the reformers. At the present day it has become clear, says Dr. Pitt, who directs the Child-Welfare Division in the League of Red Cross Societies, that child welfare is concerned with the whole of the rejuvenating life cycle. The new individual develops through the period of pregnancy to birth, infancy, young childhood, puberty, adolescence, and so to maturity and parenthood, when a new conception is initiated. Each phase requires its special precautions against disease, its special measures for ensuring a healthy passage to the next stage, and Dr. Pitt, writing in the *International Journal of Public Health*, maintains there must be an ante-natal clinic for the period from conception to birth; a maternity hospital for the accouchement; an infant consultation; a crèche; a chambre d'allaitement of a room in charge of a nurse, set apart at or near factories, to which nursing mothers can retire to feed their babies and where pregnant women can rest from work in the later stages; school and nutrition clinics; special classes for elder girls at the pre-parental age; schools for mothers at the parental age; dispensaries and hospitals for the sick, whether children or mothers. Elaborate as all this sounds, it would be hard to say which branch of the work can be safely dispensed with. The weak point in the system as commonly seen in practice in this country is the entire neglect of any attempt to prepare girls after leaving school for the duties of motherhood and wifehood, and the corresponding neglect of these ignorant creatures after they marry. The right kind of knowledge to impart to them is still a disputed matter. It might be thought that societies such as the Young Women's Christian Association and the Girls' Friendly Society might take the lead in this very interesting direction, and we believe that they might do much. Until the direction of the home and the upbringing of the children is regarded as a skilled career, and all women without exception are seriously prepared for it, child welfare will be thwarted by the person clearly responsible for carrying out the entire cycle to wit, the mother.

